# Protein-Amino Acid Metabolism Disarrangements: The Hidden Enemy of Chronic Age-Related Conditions

**DOI:** 10.3390/nu10040391

**Published:** 2018-03-22

**Authors:** Evasio Pasini, Giovanni Corsetti, Roberto Aquilani, Claudia Romano, Anna Picca, Riccardo Calvani, Francesco Saverio Dioguardi

**Affiliations:** 1 Scientific Clinical Institutes Maugeri, IRCCS Lumezzane, Cardiac Rehabilitation Division, 25065 Lumezzane (Brescia), Italy; evpasini@gmail.com; 2 Division of Human Anatomy and Physiopathology, Department of Clinical and Experimental Sciences, University of Brescia, Viale Europa, 11-25124 Brescia, Italy; cla300482@gmail.com; 3 Department of Biology and Biotechnology, University of Pavia, 27100 Pavia, Italy; dottore.aquilani@gmail.com; 4 Department of Geriatrics, Neurosciences and Orthopaedics, Catholic University of the Sacred Heart, 00198 Rome, Italy; anna.picca1@gmail.com (A.P.); riccardo.calvani@gmail.com (R.C.); 5 Department of Clinical Sciences and Community Health, University of Milan, 20122 Milan, Italy; fsdioguardi@gmail.com

**Keywords:** protein metabolism, sarcopenia, muscle wasting, amino acids, catabolism, inflammation

## Abstract

Proteins are macro-molecules crucial for cell life, which are made up of amino acids (AAs). In healthy people, protein synthesis and degradation are well balanced. However, in the presence of hypercatabolic stimulation (i.e., inflammation), protein breakdown increases as the resulting AAs are consumed for metabolic proposes. Indeed, AAs are biochemical totipotent molecules which, when deaminated, can be transformed into energy, lipids, carbohydrates, and/or biochemical intermediates of fundamental cycles, such as the Krebs’ cycle. The biochemical consequence of hyper-catabolism is protein disarrangement, clinically evident with signs such as sarcopenia, hypalbuminemia, anaemia, infection, and altered fluid compartmentation, etc. Hypercatabolic protein disarrangement (HPD) is often underestimated by clinicians, despite correlating with increased mortality, hospitalization, and morbidity quite independent of the primary disease. Simple, cheap, repeatable measurements can be used to identify HPD. Therefore, identification and treatment of proteins’ metabolic impairment with appropriate measurements and therapy is a clinical strategy that could improve the prognosis of patients with acute/chronic hypercatabolic inflammatory disease. Here, we describe the metabolism of protein and AAs in hypercatabolic syndrome, illustrating the clinical impact of protein disarrangement. We also illustrate simple, cheap, repeatable, and worldwide available measurements to identify these conditions. Finally, we provide scientific evidence for HPD nutritional treatment.

## 1. Metabolism of Proteins and Amino Acids: Essential Cellular Blocks 

Proteins are macronutrients crucial for various cellular activities, as well as body metabolism. Protein synthesis is primarily controlled by amino acid (AA) availability in stoichiometric quantities proportional to the number of proteins needed for synthesis and energy requirements needed to sustain the synthetic process. 

**Figure 1:**
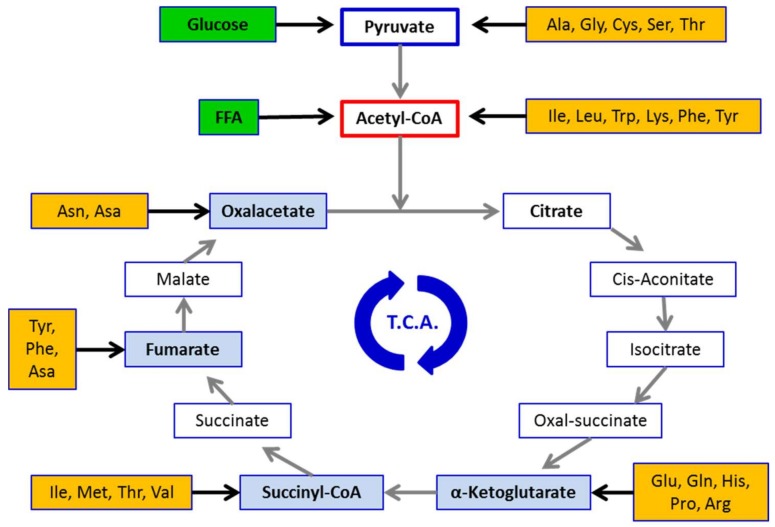
Amino acid-derived intermediates fueling the tri-carboxilic acid cycle (TCA) (Kreb’s cycle). FFA = free fatty acids. Ala = alanine, Arg = arginine, Asa = aspartic acid, Asn = asparagine, Cys = cysteine, Gln = glutamine, Glu = glutamic acid, Gly = glycine, His = histidine, Ile = isoleucine, Leu = Leucine, Lys = lysine, Met = methionine, Phe = phenylalanine, Pro = proline, Ser = serine, Thr = threonine, Trp = tryptophan, Tyr = tyrosine, Val = valine.

**Figure 2:**
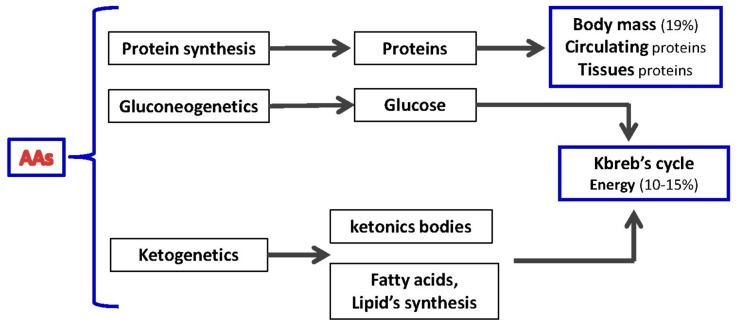
Amino acids (AAs) as biochemical totipotent molecules.

AAs serve many functions within the body. Being the only source of nitrogen for mammals, AA-derived nitrogen is pivotal for synthetizing precursors (purine and/or pyrimidine) of major energy molecules (i.e., ATP, ADP, IMP) and/or nucleic acids (i.e., DNA/RNA), and/or to produce compounds that can regulate major biochemical signaling pathways, such as nitric oxide (NO). Moreover, deamination of AAs released from skeletal muscle and/or circulating visceral proteins generate a carbon skeleton rich in oxygen and hydrogen suitable for subsequent biochemical transformation. This carbon skeleton can be used by the liver to produce glucose through gluconeogenesis and other macromolecules, such as lipids. The AA-derived carbon skeleton is also relevant in producing intermediaries fueling the Kreb’s cycle that are thereafter transformed into energy and/or other metabolic intermediaries (Figure 1). Therefore, AAs can be considered “biochemical totipotent molecules” able to be converted into energy, carbohydrates, lipids, and biochemical intermediates, dependent on body metabolic demands [1,2] (Figure 2).

From a nutritional point of view, AAs are categorized as either “non-essential” (NEAAs) or “essential” (EAAs). NEAAs are synthetized within the body from carbohydrates and lipids deriving nitrogen from other AAs. EAAs, however, cannot be synthesized and need to be adequately introduced with the diet, and they are also the most relevant nutritional input for protein synthesis [3]. For instance, leucine is considered the primary nutritional regulator of muscle protein anabolism [4] due to its ability to trigger the mammalian target of the rapamycin (mTOR) pathway and inhibit the proteasome system [4].

Interestingly, under conditions such as injury, surgery, or chronic diseases, there is increased demand for AAs as a consequence of higher resting energy expenditure [5]. The consumption of EAAs into the Kreb’s cycle and its competition with the oxidation of glucose or of fatty acids via β oxidation has been suggested as a strategy to maintain efficient energy production in pathological conditions. This is due to fat oxidation being less energy efficient than glycolysis and to the entry of AA-derived pyruvate into the mitochondrial Kreb’s cycle [6]. 

Indeed, β oxidation, which is mostly cytoplasmic, reduces the ratio of ATP/available O_2,_ and obliges large amounts of EAAs to be used as intermediates of the Kreb’s cycle. Interestingly, EAAs are used as substitutes for pyruvate-derived oxaloacetate shortened by the large amounts of NADH produced out of mitochondria due to β-oxidation [7]. 

Such a metabolic shift is one of the main alterations leading to an imbalance between nitrogen request and nitrogen intake observed in patients with chronic altered metabolic conditions and measured as the nitrogen balance. This ultimately triggers muscle and circulating protein disarrangement that become clinically evident in several muscle-wasting conditions (e.g., sarcopenia and cachexia) and/or hypoalbuminemia with or without anemia [8].

## 2. The Pathogenesis of Protein Disarrangement: The Hypermetabolic Syndrome 

The pathophysiology of syndromes characterized by protein disarrangement is multi-factorial and dishomogeneous. Indeed, both the elderly and patients with chronic diseases (such as infections and sepsis) show a pattern of circulating mediators with altered ratios between catabolic molecules (e.g., TNF-α, cortisol, catecholamines, glucagons, cytokines) inducing protein degradation and anabolic factors (e.g., insulin, insulin-like growth factors, and growth hormone) that stimulate protein synthesis. Increased catabolic stimuli and the consequent impaired anabolic/catabolic stimulation is a condition that can be referred to as the “hypercatabolic syndrome” (HS). This severely impacts whole-body metabolism and causes an imbalance between nutritional input and synthetic/energy needs [9,10]. 

**Figure 3:**
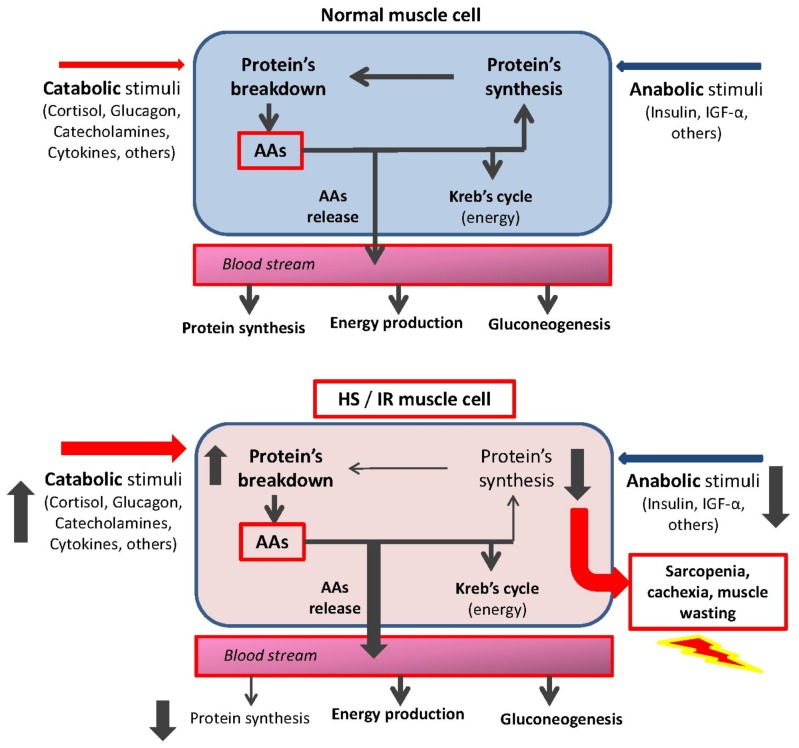
The fate of amino acids (AAs) in muscle cell: physiologic (**top**) and hypercatabolic syndrome (HS) and/or insulin resistance (IS) (**bottom**). The increase in catabolic stimuli enhances protein breakdown and AA release in the blood stream. These AAs are used almost exclusively for energy production and gluconeogenesis, but not for de novo protein synthesis. This favors the onset and aggravation of muscle wasting.

Muscle contractile proteins and circulating visceral proteins are the major reservoir of AAs within the body. Indeed, these proteins can be degraded by catabolic stimuli and/or physical exercises [11] and other AAs can be re-used by cells for de novo protein synthesis. However, a large amount of AAs are deaminated to produce energy and other metabolic intermediates via the Kreb’s cycle and/or are released into the blood stream to maintain a ready-to-use pool of AAs [10] (Figure 3). In this context, the role of skeletal muscle and circulating visceral proteins goes well beyond that of ensuring posture maintenance and locomotion and transporting molecules or atoms. 

The proteins of skeletal muscle and the circulatory system are in continuous turnover. Several studies indicate that about 250–350 g of proteins per day are metabolized in healthy individuals. However, this amount increases dramatically under conditions with higher metabolic demand. For instance, aged muscles have reduced anabolic response to low doses (e.g., less than 10 g) of EAAs [12]; yet, higher doses (e.g., 10–15 g, with at least 3 g of leucine) are sufficient to induce a protein anabolic response comparable to that observed in younger adults [12]. Therefore, it is recommended that older people consume food rich in high-quality proteins with higher proportions of EAAs, such as lean meat and other leucine-rich foods (e.g., soybeans, peanuts, chickpeas, and lentils) [13].

Recently, protein intake above the current recommended dietary allowance (RDA; 0.8 g/kg/day) has been proposed to preserve muscle health in later life [14,15,16,17]. It, therefore, appears appropriate to promote protein intake of 1.0–1.2 g/kg/day, while 1.2–1.5 g/kg/day of protein may be required in older adults with acute or chronic conditions [16,17,18]. Finally, older people with severe illnesses or overt malnutrition may need as much as 2.0 g/kg/day of protein [17]. 

It is also important to consider that HS induces “insulin resistance” (IR), a condition which reduces cytoplasmic and mitochondrial cell protein synthesis and impaired cell metabolism. This reinforces the protein-amino acid disarrangement [10,19].

## 3. Clinical Impact of Protein Disarrangements 

Alterations in protein balance have been associated with muscle wasting in patients aged 65+, hospitalized for a variety of chronic disease conditions [20]. Furthermore, approximately 30% of patients with chronic heart failure exhibit reduced serum albumin (<3.5 g/dL) [21]. Notably, these conditions are related to increased morbidity, hospitalization, and mortality, independent of primary diseases, and so increases health-related costs and worse prognosis [11,22]. 

The central role of muscle proteins for the maintenance of whole-body metabolism, especially in response to stress (e.g., HS following chronic disease conditions) has recently gained support [23]. Indeed, the maintenance of muscle mass and protein metabolism has been suggested as being a relevant parameter to include in future studies because of its clinical relevance [23]. 

Therefore, the concept of dietary protein intake being calculated as a fixed and limited ratio (<20%) of total calories has also been questioned. Two major points should be considered: (1) nitrogen needs may significantly increase independent of caloric demand. This is often overlooked in large populations (i.e., the elderly with or without chronic diseases); and (2) nitrogen should not be calculated by using total protein nitrogen content, but by considering individual AAs. This is because dietary proteins are enriched in NEAAs, which are not fundamental to support global metabolism but do increase urea synthesis, and EAAs, which are crucial for refuelling proteins and global metabolism. Thus, insufficient intake of EAAs despite increased need may be a mechanism in obese patients with chronic disease (i.e., heart failure), further worsening protein metabolism [24]. 

Dietary intake providing adequate protein amount and, consequently, AAs to match organ demand, preserves the integrity of organs essential for life, as witnessed by peripheral muscle homeostasis and, ultimately, patient survival. Conversely, insufficient EAA intake induces muscle and circulating visceral protein degradation to release AAs to cope for this deficit. Thus, sarcopenia/muscle wasting and hypoalbuminemia become clinically evident. 

HS also reduces the appetite, as well as nausea and digestive disorders in patients with chronic conditions leading to inadequate nutrition and consequent reduced availability of nutrients, including AAs [25]. Anamnesis would suggest that eating-related disorders can be found in chronic patients and specific therapeutic strategies can be implemented. Up to 50% of patients with severe chronic disease show altered protein metabolism, which is often underestimated by clinicians despite influencing cell life and having relevant clinical implications [25]. 

**Figure 4:**
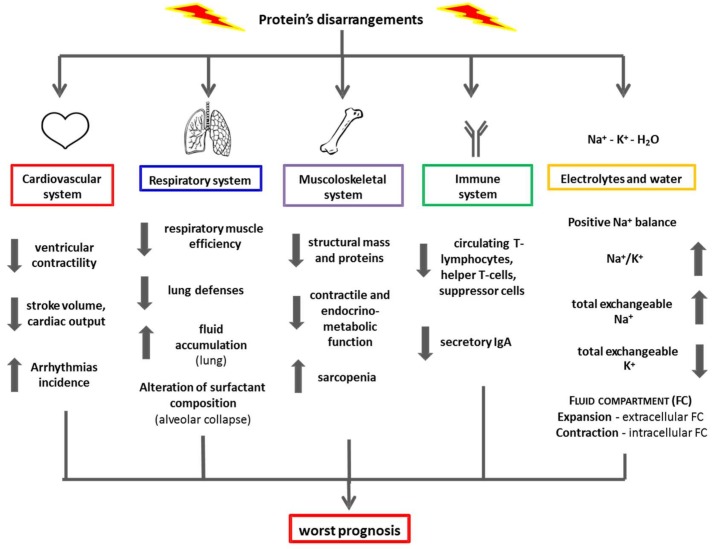
Effects of protein disarrangements on various systems and electrolyte balance.

The consequences of protein disarrangement in various body organs and/or systems are illustrated in Figure 4. Altered protein metabolism in patients with chronic diseases, especially older ones, may increase the risk of developing life-threatening complications (e.g., infection due to reduced or circulating T cells and secretion of protein Ig or imbalance of the Na^+^/K^+^ ratio) with consequent water retention, respiratory failure, and pulmonary edema. Furthermore, cardiac dysfunction, ventricular arrhythmias, and renal insufficiency may also occur [25].

## 4. Clinical Steps to Evaluate Protein Disarrangement

We have recently proposed a panel of practical and inexpensive tools for the clinical evaluation of protein disarrangement [26]. A set of indirect measurements evaluating body composition (anthropometric parameters), visceral protein composition (serum albumin, pre-albumin, transferrin, retinol binding protein, nitrogen balance), muscle protein degradation (serum or urinary excretion of 3-metil histidine), and immuno-competence (total lymphocyte count) has been proposed. However, a rapid, inexpensive, and easy assessment of protein disarrangements at the bedside is still lacking. As such, the evaluation of anthropometric parameters should be considered [26].

A simple way to evaluate body composition is to measure tricipital skin-fold thickness (TST, an index of fat mass) and arm muscle area (AMA, an index of lean mass) as described elsewhere [27,28]. Notably, TST and AMA are not modified by extracellular fluids, so they are useful tools even in patients with fluid retention. Interestingly, the presence of reduced AMA less than 5th percentile by age and sex, together with hypoalbuminemia, in the absence of liver and/or renal insufficiency, confirms the presence of muscle sarcopenia and altered protein metabolism. [29]

Whenever protein disarrangement associated with muscle wasting and hypoalbuminemia is suspected, the following additional evaluations can be considered.

### 4.1. Circulating Visceral Proteins

The concentration of serum proteins such as albumin, pre-albumin, transferrin, and retinol binding proteins are influenced by extra-cellular fluid composition.

Albumin concentration can be included in routine clinical blood measurements as it is easy to measure, non-invasive, and a repeatable marker. Its concentration correlates with worsening morbidity and mortality independent of the disease index [30]. Albumin half-life in circulation is about 20 days and the fractional replacement rate is 10% per day. In the absence of chronic stress, increased serum albumin levels appear within 14 days and a serum concentration <3.5 g/dL indicates impaired protein metabolism associated with muscle wasting [30]. Concentrations lower than 3.2 g/dL suggest more pronounced protein metabolism disarrangement. Notably, severe nephrosis, protein-losing enteropathy, or severe liver insufficiency reduce serum albumin concentrations. Consequently, these conditions should be excluded when albumin is used as an index of protein status.

Pre-albumin responds quickly to short-term (24–36 h) energy restriction and re-feeding. Repeated measurements of pre-albumin levels over a week could be a useful measure of both protein depletion and repletion. This is particularly useful to monitor treatment [30].

Transferrin correlates with mortality and is also influenced by iron metabolism. It has a half-life of about eight days and can, therefore, be used to monitor the effects of specific intervention. 

Retinol-binding protein has a turnover of 12 h. Consequently, it is a measure of rapid protein metabolism modification.

### 4.2. Nitrogen Balance 

Nitrogen balance (NB) is an indirect measure of dynamic processes of endogenous protein synthesis (anabolism) and demolition (catabolism). NB is expressed as: NB g/day = N_I_ − N_V_ + 2 g. This formula includes nitrogen intake/supply in g/day (N_I_) and urinary nitrogen excretion (N_V_) in g/day + 20% N_V_ for non-urea N excretion. Two grams correspond to the nitrogen lost in feces and sweat.

NB is in equilibrium if its balance equals ± 1 g/day. NB > 1 g/day indicates prevalent protein synthesis. NB < 1 g/day suggests ongoing protein degradation with AAs used for general metabolic purpose instead of protein synthesis. Therefore, NB < 1 g/day represents an index of proteins disarrangement [8,26]. Notably, NB depends on urea excretion that is influenced by the daily amount of NEAAs that are routed to urea excretion. To obtain reliable information, this parameter should be monitored frequently. 

### 4.3. 3-Methylistidine

Methylation of histidine is a marker of protein degradation derived from contractile actin and myosin. A simple and rapid method to estimate the fractional catabolic rate of myofibrillar protein is the evaluation of 3-methylhistidine: creatinine excretion in the urine. The presence of 3-MeH shows the presence of proteolysis over the immediate period-hours [8,31,32].

### 4.4. Blood Lymphocyte Count

The loss of circulating cell-mediated immune competence is present in patients with advanced heart failure with sarcopenia and metabolism disarrangements. Indeed, the lymphocyte count could be considered an indirect index of cell proliferation, protein synthesis, and energy availability and can be used to confirm protein and global metabolic impairment [33]. 

## 5. Possible Therapeutic Interventions 

Protein intake and AA availability are key to maintaining protein synthesis in living organisms. Healthy individuals absorb AAs from the diet after protein digestion by pancreatic enzymes. However, the pancreas uses large amounts of AAs and energy to produce digestive enzymes [10]. The efficiency of pancreatic and mesenteric circulation may be progressively reduced in HS and/or in chronic diseases with water retention [34]. In addition, gut microbiota alterations and impaired intestinal function, including altered nutrient digestion and absorption, have been reported in patients with chronic disease [34]. These conditions lead to impaired AA digestion and absorption and, consequently, to reduced AA plasma levels that may become insufficient to maintaining protein synthesis and energy demand in HS patients [10]. In contrast, individual AAs derived from nutritional supplements are immediately available after absorption and transit into the bloodstream to be delivered to cells [35]. 

EAAs stimulate protein synthesis in both young and the elderly [36]. However, it has recently been shown that specific diets containing blends of individual EAAs in stoichiometric ratios are crucial for providing AAs for various metabolic needs, such as protein synthesis, mitochondrial biogenesis, and other important metabolic pathways crucial for cell life [6,37,38]. Indeed, specific EAA mixtures control protein synthesis in myocytes by activating AMP-activated protein kinase (AMPK) and mTOR, which regulate energy production/use, protein synthesis, cell proliferation, mitochondrial biogenesis, and anti-apoptotic process [39].

Clinical and experimental data suggest that oral supplements with specific individual EAA mixtures ensuring metabolic energy supply, administered traditionally, counteract protein disarrangement and cellular energy impairment without influencing renal function [40,41,42,43]. The clinical consequences of this is that the percentage of nitrogen and calories provided by diet should be calculated separately according to metabolic needs. Moreover, the amount of EAAs should be provided as a function of their intrinsic capacities to maintain proteins and body metabolism. In addition, individual AAs should be provided so they are not rapidly absorbed, thus increasing their blood viability [44]. 

Taken together, these observations could explain why previous studies were unable to show any effects of simple total protein dietary supplementation on protein and energy metabolism in patients with chronic diseases [45]. Such findings support the indication that the elderly need increased EAA to stimulate muscle protein synthesis. This also introduces the concept of evaluating protein AA composition (protein quality) [46]. A modification of the Dietary Guidelines of Americans (DG of A), which provides nutrient advice to avoid/reduce age-related nutritional problems, has been proposed as follows: (1) protein (and more importantly specific AAs) should be a part of the adult/aged people diet; (2) protein (and more importantly specific AAs) needs for adult/aged people should be proportional to body weight and/or clinical condition and not as a percentage of total energy intake; (3) most adult/aged people benefit from protein intake above the minimum recommended daily allowance. Indeed, to maintain healthy muscles and bones, at least 30 g of high-quality protein (and more importantly specific EAAs) should be ingested at more than one meal.

The effect of dietary administration of different EAAs blends has been actively investigated in the recent years [42,43,44]. Existing findings on the molecular pathways elicited by proteins and AA metabolism in chronic disease conditions could allow the development of therapeutic strategies to contrast metabolic impairment, especially in the elderly. 

## 6. Conclusions

The evaluation of protein disarrangement deserves greater attention to manage chronic diseases, especially in old age (see Box 1). Readily available and inexpensive anthropometric and blood parameters, such as TST, AMA, and albuminemia, can be obtained routinely at the bedside. Additional research could unveil the causes of these conditions and monitor the outcome of specific therapeutic interventions.

Clinicians should consider protein homeostasis as essential to maintaining metabolic competence in patients with chronic diseases. This is fundamental for any further therapeutic approaches. The identification and treatment of protein metabolic impairment with appropriate therapies may be at least as important as any other evaluation and therapeutic strategy implemented in improving the prognosis of chronically ill patients.

Box 1 **Main Messages from the text.**

- Proteins are building blocks for living organisms consisting of amino acids (AAs).
- AAs are totipotent biochemical molecules essential for cellular activity.
- Pathological conditions increase protein/AA demand.
- Hypercatabolic syndromes, due to increased inflammation and catabolic hormones, cause significant changes in body metabolism and lead to an imbalance between nutritional input and synthetic/energy needs.
- Both circulating (i.e., albumin) and muscle proteins are a reservoir of AAs (above all, essential AAs) within the body.
- Altered protein metabolism in patients (especially those with chronic diseases and/or older), can increase the risk of developing life-threatening complications (e.g., infection, cardiac and/or renal dysfunction).
- The pathophysiology of conditions characterized by protein disarrangements (i.e., muscle wasting and/or hypoalbuminemia) need to be fully clarified to develop adequate therapeutic (nutritional) strategies.
- Clinicians need to consider protein metabolism as a critical aspect for the management of patients with chronic diseases.

## References

[1] Lehninger A.L. (1982). Principles of Biochemistry.

[2] Bischoffa R., Hartmut S. (2012). Amino acids: Chemistry, functionality and selected non-post-translational modifications. J. Proteom..

[3] Mitchell H.H., Block R.J. (1946). Some relationships between the amino acid contents of proteins and their nutritive values for the rat. J. Biol. Chem..

[4] Anthony J.C., Anthony T.G., Kimball S.R., Jefferson L.S. (2001). Signalling pathways involved in translational control of protein synthesis in skeletal muscle by leucine. J. Nutr..

[5] Woolfson A.M.J. (1983). Amino acids-their role as an energy source. Proc. Nutr. Soc..

[6] Dioguardi F.S. (2004). Wasting and the substrate to energy controller pathway: A role for insulin resistance and amino acids. Am. J. Cardiol..

[7] Taegtmayer H. (1994). Energy metabolism of the heart: From basic concepts to clinical applications. Curr. Prob. Cardiol..

[8] Aquilani R., Opasich C., Verri M., Boschi F., Febo O., Pasini E., Pastoris O. (2003). Is nutritional intake adequate in chronic heart failure patients?. J. Am. Coll. Cardiol..

[9] Anker S.D., Chaua T.P., Ponikowski P., Harrington D., Swan J.W., Kox W.J., Poole-Wilson P.A., Coats A.J. (1997). Hormonal changes and catabolic/anabolic imbalance in chronic heart failure and their importance for cardiac cachexia. Circulation.

[10] Pasini E., Aquilani R., Dioguardi F.S., D’Antona G., Gheorghiade M., Taegtmeyer H. (2008). Hypercatbolic syndrome: Molecular basis and effects of nutritional supplementation with amino acids. Am. J. Cardiol..

[11] Aquilani R., Opasic C., Dossena M., Iadarola P., Gualco A., Arcidiaco P., Viglio S., Boschi F., Verri M., Pasini E. (2005). Increased skeletal muscle amino acid release with light exercise in deconditioned patients with heart failure. J. Am. Coll. Cardiol..

[12] Katsanos C.S., Kobayashi H., Sheffield-Moore M., Aarsland A., Wolfe R.R. (2005). Aging is associated with diminished accretion of muscle proteins after the ingestion of a small bolus of essential amino acids. Am. J. Clin. Nutr..

[13] Martone A.M., Marzetti E., Calvani R., Picca A., Tosato M., Santoro L., Di Giorgio A., Nesci A., Sisto A., Santoliquido A., Landi F. (2017). Exercise and Protein Intake: A Synergistic Approach against Sarcopenia. Biomed. Res. Int..

[14] Fielding R.A., Vellas B., Evans W.J., Bhasin S., Morley J.E., Newman A.B., Abellan van Kan G., Andrieu S., Bauer J., Breuille D. (2011). Sarcopenia: An undiagnosed condition in older adults. Current consensus definition: Prevalence, etiology, and consequences. International working group on Sarcopenia. J. Am. Med. Dir. Assoc..

[15] Volpi E., Campbell W.W., Dwyer J.T., Johnson M.A., Jensen G.L., Morley J.E., Wolfe R.R. (2013). Is the optimal level of protein intake for older adults greater than the recommended dietary allowance?. J. Gerontol. A Biol. Sci. Med. Sci..

[16] Morley J.E., Argiles J.M., Evans W.J., Bhasin S., Cella D., Deutz N.E.P., Doehner W., Fearon K.C.H., Ferrucci L., Hellerstein M.K. (2010). Society for Sarcopenia, Cachexia, and Wasting Disease. Nutritional recommendations for the management of Sarcopenia. J. Am. Med. Dir. Assoc..

[17] Bauer J., Biolo G., Cederholm T., Cesari M., Cruz-Jentoft A.J., Morley J.E., Phillips S., Sieber C., Stehle P., Teta D. (2013). Evidence-based recommendations for optimal dietary protein intake in older people: A position paper from the PROT-AGE Study Group. J. Am. Med. Dir. Assoc..

[18] Paddon-Jones D., Short K.R., Campbell W.W., Volpi E., Wolfe R.R. (2008). Role of dietary protein in the Sarcopenia of aging. Am. J. Clin. Nutr..

[19] Tremblay F., Lavigne C., Jacques H., Marette A. (2007). Role of Dietary Proteins and Amino Acids in the Pathogenesis of Insulin Resistance. Annu. Rev. Nutr..

[20] Guigoz Y. (2006). The mini nutritional assessment review of the literature: What does it tell us?. J. Nutr. Health Aging.

[21] Liu M., Chan C.P., Yan B.P., Zhang Q., Lam Y.Y., Li R.J., Sanderson J.E., Coats A.J., Sun J.P., Yip G.W., Yu C.M. (2012). Albumin levels predict survival in patients with heart failure and preserved ejection fraction. Eur. J. Heart Fail..

[22] Anker S.D., Ponikowski P., Varney S., Chua T.P., Clark A.L., Webb-Peploe K.M., Harrington D., Kox W.J., Poole-Wilson P.A., Coats A.J. (1997). Wasting as independent risk factor for mortality in chronic heart failure. Lancet.

[23] Wolfe E.W. (2006). The underappreciated role of muscle in health and diseases. Am. J. Clin. Nutr..

[24] Lainscak M., von Haehling S., Doehner W., Anker S.D. (2012). The obesity paradox in chronic disease: Facts and numbers. J. Cachexia Sarcopenia Muscle.

[25] Aquilani R., Opasich C., Viglio S., Iadarola P., Pasini E. (2008). Nutrition in acute decompensation of patients with acute heart failure syndrome. Acute Heart Failure.

[26] Pasini E., Aquilani R., Dioguardi F.S. (2013). The enemy within. How to identify chronic diseases induced-protein metabolism impairment and its possible pharmacological treatment. Pharmacol. Res..

[27] Magnani R. (1997). Sampling guide. Food and Nutrition Technical Assistance (FANTA) Project.

[28] Cogil B. (2003). Anthropometric indicators measurement guide. Food and Nutrition Technical Assistance (FANTA) Project.

[29] Frisancho R. (1990). Anthropometric Standards for the Assessment of Growth and Nutritional Status.

[30] Watson R.R., Watson R.R. (1984). Nutritional stresses: Levels of complement proteins and their functions. Nutrition, Disease Resistance and Immune Function.

[31] Ferrari F., Fumagalli M., Viglio S., Aquilani R., Pasini E., Iadarola P. (2009). A rapid method for simultaneous determination of creatine, 1-and 3 methyhistidine in human urine. Electrophoresis.

[32] Aquilani R., Opasic C., Gualco A., Bairdi P., Pasini E., Testa A., Viglio S., Iadarola P., Verri M., D’Agostino L., Boschi F. (2009). A practical method to diagnose muscle degradation in normo-nourished patient with chronic heart failure. Int. J. Med..

[33] Acanfora D., Gheorghiade M., Trojano L., Furgi G., Pasini E., Picone C., Papa A., Iannuzzi G.L., Bonow R.O., Rengo F. (2001). Relative lymphocyte count: A prognostic indicator of mortality in elderly patients with congestive heart failure. Am. Heart J..

[34] Pasini E., Aquilani R., Testa C., Baiardi P., Angioletti S., Boschi F., Verri M., Dioguardi F.S. (2016). Pathogenic Gut Flora in Patients with Chronic Heart Failure. JACC Heart Fail..

[35] Rondanelli M., Aquilani R., Verri M., Boschi F., Pasini E., Perna S., Faliva A., Condino A.M. (2017). Plasma kinetics of essential amino acids following their ingestion as free formula or as dietary protein components. Aging Clin. Exp. Res..

[36] Volpi E., Kobayashi H., Sheffield-Moore M., Mittendorfer B., Wolfe R.R. (2003). Essential amino acids are primarily responsible for the amino acid stimulation of muscle protein anabolism in healthy elderly adults. Am. J. Clin. Nutr..

[37] Nisoli E., Cozzi V., Carruba M. (2008). Amino Acids and mitochondrial biogenesis. Am. J. Cardiol..

[38] D’Antona G., Ragni M., Cardile A., Tedesco L., Dossena M., Bruttini F., Caliaro F., Corsetti G., Bottinelli R., Carruba M.O. (2010). Branched-chain amino acid supplementation promotes survival and supports cardiac and skeletal muscle mitochondrial biogenesis in middle-aged mice. Cell Metab..

[39] Fujita S., Dreyer H.C., Drummond M.J., Glynn E.L., Cadenas J.G., Yoshizawa F., Volpi E., Rasmussen B.B. (2007). Nutrient signalling in the regulation of human muscle protein synthesis. J. Physiol..

[40] Aquilani R., Viglio S., Idarola P., Opasich C., Testa A., Dioguardi F.S., Pasini E. (2008). Oral amino acid supplementation improve exercise capacities in elderly patients with heart failure. Am. J. Cardiol..

[41] Scognamiglio R., Testa A., Aquilani R., Dioguardi F.S., Pasini E. (2008). Impairment in walking capacity and myocardial function in the elderly: It is a role for non-pharmacologic therapy with nutritional amino acid supplementation?. Am. J. Cardiol..

[42] Aquilani R., Opasich C., Gualco C., Veiir M., Testa A., Pasini E., Viglio C., Iadarola P., Pastoris O., Dossena M. (2008). Adequate energy-protein intake is not enough to improve nutritional and metabolic status in muscle-depleted patients with chronic heart failure. Eur. J. Heart Fail..

[43] Solerte S.B., Gazzaruso C., Bonacasa R., Rondanelli M., Zamboni M., Basso C., Locatelli E., Schifino N., Giustina A., Fioravanti M. (2008). Nutritional supplements with oral amino acid misture increases whole body lean mass and insulin sensitivity in elderly subjects with Sarcopenia. Am. J. Cardiol..

[44] Corsetti G., Pasini E., D’Antona G., Nisoli E., Flati V., Assanelli D., Dioguardi S.F., Bianchi R. (2008). Morphometric changes induced by amino acid supplementation in skeletal and cardiac muscles of old mice. Am. J. Cardiol..

[45] Broqvist M., Dahlstrom U., Larsson J., Larsson J., Nylander E., Permert J. (1994). Nutritional assessment and muscle energy metabolism in severe chronic congestive heart failure: Effects of long-term dietary supplementation. Eur. Heart J..

[46] Layman D.K. (2009). Dietary Guidelines should reflect new understandings about adult protein needs. Nutr. Metab..

